# Identification of a Homozygous *PEX26* Mutation in a Heimler Syndrome Patient

**DOI:** 10.3390/genes12050646

**Published:** 2021-04-26

**Authors:** Youn Jung Kim, Yuichi Abe, Young-Jae Kim, Yukio Fujiki, Jung-Wook Kim

**Affiliations:** 1 Department of Molecular Genetics, School of Dentistry & DRI, Seoul National University, Seoul 03080, Korea; ykim71@snu.ac.kr; 2 Faculty of Arts and Science, Kyushu University, Fukuoka 819-0315, Japan; abe.yuichi.439@m.kyushu-u.ac.jp; 3 Department of Pediatric Dentistry, School of Dentistry & DRI, Seoul National University, Seoul 03080, Korea; neokarma@snu.ac.kr; 4 Institute of Rheological Functions of Food, Fukuoka 811-2501, Japan

**Keywords:** amelogenesis imperfecta, hearing loss, whole exome sequencing, PEX26, temperature sensitivity, Heimler syndrome

## Abstract

This study aimed to identify the molecular genetic etiology of an 8-year-old boy with amelogenesis imperfecta in permanent dentition. Bilateral cochlear implants were placed due to sensorineural hearing loss, and there was no other family member with a similar phenotype. Peripheral blood samples were collected with the understanding and written consent of the participating family members. A constitutional chromosome study was performed for the proband. Genomic DNA was isolated, and whole exome sequencing was performed. A series of bioinformatic analyses were performed with the obtained paired-end sequencing reads, and the variants were filtered and annotated with dbSNP147. There was no abnormality in the constitutional chromosome study. Whole exome sequencing analysis with trio samples identified a homozygous mutation (c.506T>C, p. (Leu169Pro)) in the *PEX26* gene. We verified **“**temperature sensitivity (*ts*)” of patient-derived Pex26-L169P by expression in *pex26* CHO mutant ZP167 cells to determine the effect of the L169P mutation on Pex26 function. The L169P mutation causes a mild *ts*-cellular phenotype representing the decreased peroxisomal import of catalase. This study supports the finding that the recessive mutations in *PEX26* are associated with Heimler syndrome and demonstrates the importance of an early and correct diagnosis.

## 1. Introduction

Amelogenesis imperfecta (AI) is a collection of hereditary enamel defects that are heterogeneous in genetic etiology and clinical phenotype [1]. Clinically, AI can be categorized as hypoplastic, hypocalcification and hypomaturation according to the characteristics of the affected enamel. In some cases, it is hard to determine the exact clinical phenotype; therefore, the hypocalcification and hypomaturation types are grouped as the hypomineralization type [2].

AI can occur as an isolated entity without non-oral symptoms or as an accompanied phenotype to a syndrome [3]. Heimler syndrome (HS, OMIM #234580 for HMLR1 and #616617 for HMLR2) is a rare autosomal recessive disorder characterized by sensorineural hearing loss, AI, nail abnormalities, and occasional or late-onset retinal pigmentation [4,5]. It has been identified that hypomorphic mutations in the peroxisome-biogenesis genes *PEX1* (OMIM *602136) and *PEX6* (OMIM *601498) cause HS [6]. Recent findings suggest that HS is caused by mutations in *PEX26* (OMIM *608666) as well [7].

In this study, we recruited a nuclear family with sensorineural hearing loss and AI in the permanent dentition and identified a homozygous mutation in the *PEX26* gene. Early molecular diagnostics as a precision medicine confirmed HS in the proband and enabled us to refer the proband to the appropriate medical personnel and manage the symptoms.

## 2. Materials and Methods

### 2.1. Human Subjects Enrollment

The study protocol was reviewed and approved by the Institutional Review Board at Seoul National University Dental Hospital. A nuclear Korean family was recruited for this genetic study. Clinical and radiological examinations were performed, and peripheral blood samples were collected with the understanding and written consent of each participant or a guardian according to the Declaration of Helsinki.

### 2.2. DNA Isolation and Whole Exome Sequencing

A constitutional chromosome study was performed for the proband. Genomic DNA was isolated from 2 mL of peripheral whole blood from the participating family members with the NucleoSpin genomic DNA purification kit (Macherey-Nagel GmbH & Co., Düren, Germany), and the quality and quantity of the purified DNA were measured. Whole exome sequencing was performed after exome capturing with the Agilent SureSelect XT Human All Exon V5 Target Enrichment System, and 101-bp paired-end sequencing reads were obtained with the Illumina HiSeq 2500 (Theragen Etex Bio Institute, Suwon-si, Korea).

### 2.3. Bioinformatic Analysis

The program Cutadapt was used to trim the adapter sequences from the obtained sequencing reads [8], and the trimmed sequence reads were aligned to the human reference genome assembly hg38 with the Burrows–Wheeler Aligner [9]. A series of bioinformatics tools [10,11,12], including Samtools, Genome Analysis Tool Kit, and Annovar, were used to obtain sequence variants including nucleotide changes and small insertions and deletions. Sequence variants were annotated with dbSNP build 147 and filtered with a cutoff value of 0.01 for the minor allele frequency (MAF). FishingCNV was used to detect copy number variation (CNV) in exome sequencing data [13].

### 2.4. Sanger Sequencing

Sanger sequencing was performed to confirm the identified variation in the *PEX26* gene (chr22(GRCh38): NG_008339.1:g.10652T>C; NC_000022.11:g.18083571T>C; NM_017929.5:c.506T>C; NP_060399.1:p.(Leu169Pro); NCBI Gene ID:55670). PCR amplifications (491 bp) were done with the HiPi DNA polymerase premix (Elpis Biotech, Daejeon, Korea) using exon 4 specific primers (forward primer: 5′-GAGGGGCTGGATAGGAGAAG-3′; reverse primer: 5′-TGCTTCTAAGCTCGCAGGAG-3′). PCR amplification products were purified with a PCR purification kit following the manufacturer’s instructions (Elpis Biotech). DNA sequencing was performed with the reverse primer at a DNA sequencing center (Macrogen, Seoul, Korea).

### 2.5. Cell Culture

*PEX26*-deficient CHO cell mutant ZP167 [14] was maintained in Ham’s F-12 medium (Invitrogen) supplemented with 10% FBS under conditions of 5% CO_2_, 95% air at 37 °C. For analysis of temperature-sensitive (*ts*) phenotype, cells were cultured at 42 °C for 2 days.

### 2.6. Transfection of PEX26

*pex26* ZP167 cells were transfected with the expression plasmids each containing pCMVSPORT-*FLAG*-*PEX26* and -*FLAG*-*PEX26-L169P* by lipofection [14]. 

### 2.7. Antibodies & Other Methods

Rabbit anti-catalase antiserum [15] and guinea pig anti-Pex14 antiserum [16] were used for cell staining. Mouse monoclonal antibody to the FLAG epitope (M2; Sigma) was purchased. Rabbit anti-Pex14 antibody [17] was used in immunoblotting. Immunoblotting and immunofluorescence microscopy were performed as described [18].

## 3. Results

**Figure 1:**
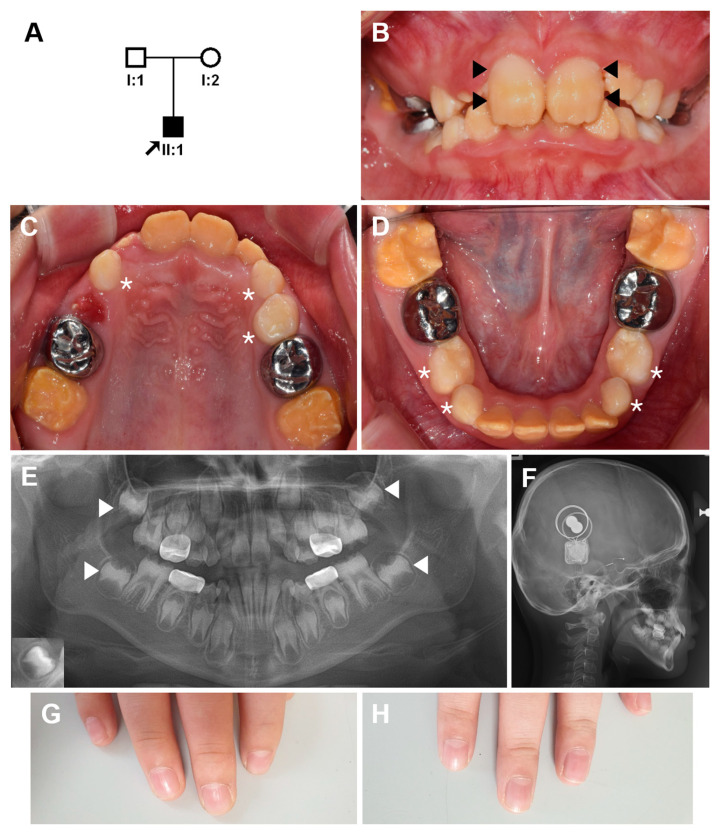
Pedigree, clinical photos, and radiographs of the proband. (**A**) Pedigree of the family. The arrow indicates the proband, and individual IDs are shown below the symbols. (**B**–**D**) Frontal, maxillary and mandibular clinical photos of the proband at age 8 years 7 months. The buccal side of the maxillary central incisors shows relatively normal looking areas in the middle and cervical thirds (black arrow heads). Deciduous teeth (white asterisks) do not have hypoplastic enamel or discoloration, but the permanent teeth show hypoplastic enamel and brown discoloration. (**E**) A panoramic radiograph revealed hypoplastic enamel in the developing permanent dentition. Almost no covering enamel can be easily seen in the second molars (white arrow heads) compared to the normal tooth in an inset at the lower left corner. (**F**) Bilateral cochlear implants can be seen in the lateral cephalogram. (**G**,**H**) His finger nails are normal.

The proband was an 8-year-old boy from a non-consanguineous family presenting with enamel hypoplasia in the permanent dentition with severe tooth sensitivity to thermal stimuli (Figure 1A). The primary dentition was reported to be normal without any symptoms. The primary second molars were treated with stainless steel crowns due to dental caries. His permanent first molars showed brown discoloration, and the enamel was very thin (Figure 1B–D). The permanent anterior teeth also showed brown discoloration and hypoplastic AI. Interestingly, the maxillary central incisors had a unique form of hypoplastic and discolored enamel. The buccal side of the teeth showed relatively normal looking areas in the middle and cervical thirds. A panoramic radiograph revealed hypoplastic enamel in the developing permanent dentition (Figure 1E).

He was born 4 weeks premature, but his weight was normal. One week after birth, he was kept in an incubator for two weeks due to a high fever. He was allergic to milk products until the age of 1 year; thus, he had been fed soy milk. His hearing was normal at birth, but gradually worsened, and he was diagnosed with progressive sensorineural hearing loss at the age of 1 year 6 months. The cochlear implant surgery was done at age 6 and 7 years, separately (Figure 1F). He has been wearing Dream Lenses at night because recently, his vision has been rapidly deteriorating bilaterally. Otherwise, there are no other symptoms, and there are no other family members with a similar phenotype (Figure 1G,H).

There was no abnormality in the constitutional chromosome study. Whole exome sequencing resulted in good mapping rates and target coverages (Table 1). CNV analysis with FishingCNV gave no candidate. PEX1 and PEX6 were further checked manually with IGV browser.

Annotated exonic and splicing variants from whole exome trio analysis were further filtered. Variants with benign prediction by Polyphen-2 were filtered. Filtered variants were selected according to the genotypes. There was no spontaneous variant. Therefore, homozygous and compound heterozygous variants were selected (Table 2).

**Figure 2:**
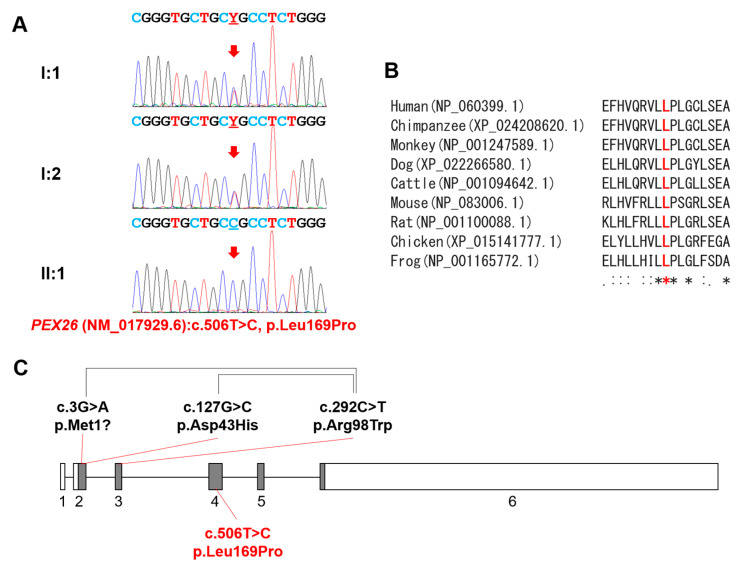
Sequencing chromatograms, homology, and gene diagram. (**A**) Sequencing chromatograms of the family members. Individual IDs are indicated on the left of each chromatogram. Nucleotide sequences are shown above the chromatograms. The mutated nucleotide is indicated by a red arrow and underlined. Y denotes mixed nucleotides of T and C. (**B**) Homology among vertebrates. Mutated amino acid is indicated by the bold and red character. Asterisks under the sequences indicate perfectly conserved amino acid positions. (**C**) Gene diagram of the *PEX26* gene (NM_017929.6). Boxes indicate the exons, and the exon numbers are shown below the boxes. Gray areas in the box indicate coding regions, and white areas indicate untranslated regions. Mutations above the diagram are previously reported compound heterozygous mutations, and paired mutations are connected with black connecting lines. The homozygous mutation identified in this study is shown below the diagram.

Selected sequence variants were further analyzed by a mutation analysis software, Alamut Visual. Variant chr1:146972960A>G was identified in the dbSNP (rs1484197674) with MAF more than 0.1. Likewise, a paternal variant chr9:66986614C>T was also removed (rs759025747). Another missense variant (chr1:146994509C>G) had prediction being tolerated by SIFT and a low CADD prediction value. Investigation of the two insertional variants (in *DACH1* and *ZNF787*) showed weak conservation with a stretch of the same amino acids near the variant positions. Therefore, the only remaining mutation with a high CADD prediction value was a homozygous mutation in the *PEX26* gene. The mother and father were carriers with no related symptoms. The identified mutation (NM_017929.6:c.506T>C) would change the leucine to proline at the 169 codon position (Figure 2A). This mutation is listed in dbSNP (rs768604587) with an extremely low allele frequency (2/251186, GnomAD_exome) and is predicted to be deleterious (with a score of 0.00), disease-causing (probability of 1) or probably_damaging (with a score of 1.000) by in silico analyses (SIFT, Mutation Taster and Polyphen-2, respectively). Moreover, homology analysis showed perfect conservation of the affected amino acid among vertebrates (Figure 2B). Gene diagram of the *PEX26* gene is shown with mutations (Figure 2C).

**Figure 3:**
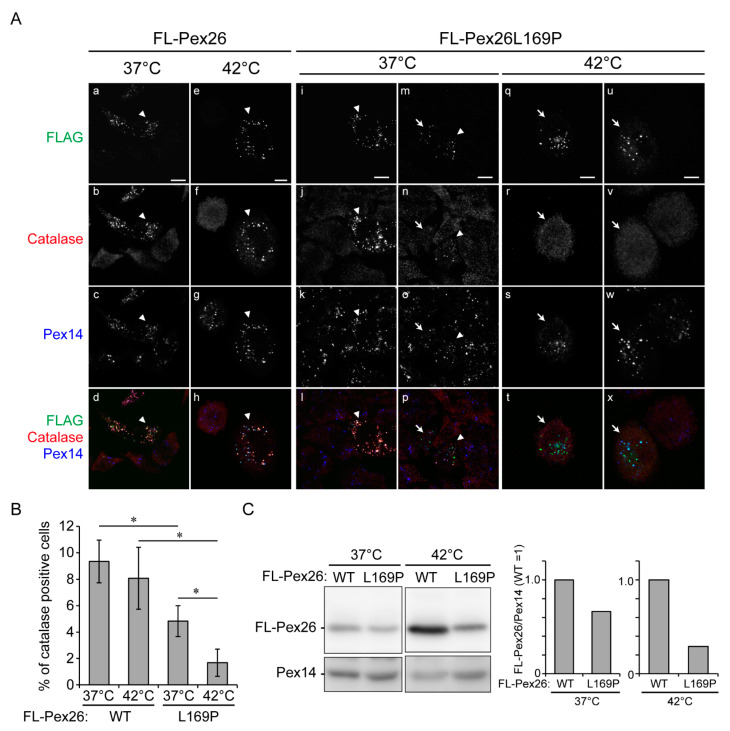
Dysfunction of Pex26-L169P. (**A**) *pex26* CHO ZP167 cells transfected with *FLAG*-tagged human *PEX26* and *PEX26-L169P* were cultured at 37 °C and 42 °C for 2 days and then stained with antibodies to FLAG (top panels: a, e, i, m, q, and u), a matrix protein catalase (middle upper panels: b, f, j, n, r, and v), and a membrane peroxin Pex14 (middle lower panels: c, g, k, o, s, and w). Merged views were also shown (bottom panels: d, h, l, p, t, and x). Scale bars, 10 µm. Arrowheads indicate FLAG-, catalase-, and Pex14-positive cells, whereas arrows show cells positive with FLAG and Pex14. (**B**) In A, catalase- and FLAG-positive cells were counted and represented as percentages of total cells counted (500 cells each, *n* = 3). Data represent means ± S.D. (*n* = 3). * *p* < 0.05; Tukey-Kramer test. (**C**) Expression level of FLAG-Pex26 and FLAG-Pex26-L169P was analyzed by SDS-PAGE of respective cell lysates and immunoblotting with anti-FLAG antibody (left). Pex14, a loading control. The amounts of both FLAG-Pex26 variants were represented by normalizing the levels of FLAG-Pex26 variants with Pex14 in respective cell lysates (right).

The functional study revealed the *ts* phenotype and decreased peroxisomal catalase import by the mutation. In FLAG-Pex26- and endogenous Pex14-expressing *pex26* CHO mutant ZP167 cells, catalase was observed as punctate-staining structures in a manner of colocalization with FLAG-Pex26 and Pex14 at 37 °C, indicative of localization in the peroxisomes (Figure 3A, a–d, arrowheads). In FLAG-Pex26-L169P- and Pex14-expressing ZP167 cells, catalase was discernible detected observed as punctate-staining, Pex14- and FLAG-Pex26-L169P-positive structures at 37 °C, suggesting that peroxisomal import of catalase was restored, but less efficiently as compared to the FLAG-Pex26 (Figure 3A, i–l, arrowheads; Figure 3B). On the other hand, in Pex26-L169P-expressing and Pex14-positive ZP167 cells (~10%), catalase was not detected in puncta, indicative of no import (Figure 3A, m–p, arrows). *ts* peroxisome assembly was reported to be responsible for the milder clinical phenotype of infantile Refsum disease (IRD) [19,20]. To assess the *ts* phenotypic property of Pex26-L169P, cells were cultured at 42 °C for 2 days. Catalase was detected mostly in a diffuse staining pattern with FLAG-Pex26-L169P- and Pex14-positive puncta at 42 °C (Figure 3A, q–x, arrows), indicating that catalase was not imported to peroxisomes at 42°C (Figure 3A,B). Expression of normal FLAG-Pex26 in *pex26* ZP167 cells restored the impaired catalase import, efficiently at 37 °C and 42 °C (Figure 3A,B). These findings suggest the significantly abrogated import of catalase in Pex26-L169P-expressing cells at 42 °C, although catalase was imported at several-fold higher level under normal culture condition at 37 °C. Expressed levels of FLAG-tagged wild-type Pex26 and Pex26-L169P in ZP167 cells were verified by immunoblotting. FLAG-Pex26-L169P was at a level lower than FLAG-Pex26 at both temperatures, suggesting that Pex26-L169P was less stable than the wild-type (Figure 3C). A point of note, is the fact that the expression of FLAG-Pex26 variants was higher at 42 °C, where catalase import was more severely affected in FLAG-Pex26-L169P-expressing in ZP167 cells, implying the inactivated restoring activity.

## 4. Discussion

Peroxisome biogenesis disorders (PBDs) are caused by a failure of peroxisome development, including its membranes, matrix, and assembly. Pathogenic mutations in at least 13 different *PEX* genes encoding peroxins are responsible for a group of PBDs and Zellweger spectrum disorders (ZSDs) [21]. ZSDs have an estimated prevalence of 1:50,000 births and consist of a spectrum of disorders from a severe to mild form: Zellweger syndrome (ZS, OMIM #214100), neonatal adrenoleukodystrophy (NALD, OMIM #601539), infantile Refsum disease (OMIM #601539), and HS [22].

PEX26, peroxisome biogenesis factor 26, recruits PEX1-PEX6 complexes to peroxisomes, and this complex is involved in the regulation of pexophagy, the autophagic degradation of peroxisomes [23]. The mutation identified in this study would result in a change of a highly conserved amino acid (Leu169) in the PEX6-binding domain (aa29-174) of the PEX26 protein [24].

We analyzed patient-derived Pex26-L169P by expression in *pex26* ZP167 cells followed by immunocytochemistry and the “temperature sensitivity” assay to determine the effect of the L169P mutation on Pex26 protein function. Earlier studies on patient-derived fibroblasts have demonstrated the association between Pex26 mutations found in patients with a clinically milder phenotype and the *ts* cellular phenotype that results from these mutations [19,20,25]. In general, fibroblasts from patients with mild ZSD have a *ts* phenotype, whereas fibroblasts with Pex26 mutations causing the severe ZS clinical phenotype are not *t*s [19].

We thus characterized the effect of the L169P mutation on Pex26 function by incubating *pex26* ZP167 cells ectopically transfected with *FLAG-PEX26-L169P* at 37 °C and 42 °C. At 37 °C, peroxisomal import of catalase appeared less efficient than the import in wild-type Pex26-expressing ZP167 cells (Figure 3A,B). Moreover, at 42 °C, FLAG-Pex26-L169P-expressing cells severely decreased catalase import as compared to those expressing wild-type FLAG-Pex26 (Figure 3A,B), indicating destabilization of Pex26 activity despite its proper membrane targeting. Interestingly, the total amount of FLAG-Pex26-L169P protein was reduced to ∼70% and ∼30% at 37 °C and 42 °C, respectively, compared to wild-type FLAG-Pex26 (Figure 3C). This indicates that the mutated protein Pex26-L169P in cells is less stable. Our results collectively show the L169P mutation causes a mild cellular phenotype representing the decreased peroxisomal protein import, implicative to the clinical phenotype of the patient with Heimler syndrome described in this report. Similar *ts*-phenotype in the catalase import was reported in fibroblasts from patients with Heimler syndrome caused by hypomorphic mutations in *PEX1* and *PEX6* [6].

HS is a rare and mild form of the ZSDs and mostly caused by mutations in the *PEX1* or *PEX6* genes [26]. Variant analysis has suggested that HS results from genotypes including milder hypomorphic alleles. HS caused by pathologic mutations in the *PEX26* gene is very rare; only one study with two patients has been reported so far [7]. Analysis of the reported HS patients confirmed that the key diagnostic feature of HS from other ZSDs is the existence of the enamel phenotype, AI [26]. It has been noted that the nail abnormality only occurred in twelve out of 31 cases; therefore, it has been suggested that nail abnormality should not be a clinical diagnostic indicator of HS [27]. We could confirm this suggestion because there was no nail abnormality in our case.

There was no consanguinity in the family; therefore, finding a homozygous mutation in the *PEX26* gene was unexpected. Given the extremely low allele frequency, it is highly possible that the mutation would be inherited from a common ancestor, identical by descent (IBD), not by coincidental mutation occurred by a mutational hotspot. The parents were checked again for any possible connection in their pedigrees including home town, but there was nothing related. The mutation could be a rare polymorphism in a Korean population.

In conclusion, we identified a homozygous *PEX26* mutation in a non-consanguineous Korean family with HS. This finding not only expands the mutational spectrum of *PEX26* mutation in HS but also confirms the suggested diagnostic features of HS. A genotype–phenotype relationship with more studies would help us to understand the normal and pathologic disease mechanism including enamel formation.

## Figures and Tables

**Table 1:** Statistics for whole exome sequencing.

Sample	Total Reads	Mapping Rate (%)	Median Target Coverage	Coverage of Target Region (%)	Fraction of Target Covered with at Least	
					20X	10X
I:1	69,365,294	99.6	95	96.5	93.5	95.5
I:2	68,921,817	99.6	87	96.3	93	95.3
II:1	59,890,807	99.7	82	96.5	92.8	95.4

**Table 2:** Filtered sequence variants.

Genomic Variant	Gene	Amino Acid Change	dbSNP	Genotype	CADD
chr1:146972960A>G	*NBPF12*	NM_001278141:p.(Lys601Glu)	.	homozygous	9.37
chr1:146994509C>G	*NBPF12*	NM_001278141:p.(Ser1436Arg)	rs202167770	homozygous	0.088
chr13:146994509insGCCGCC	*DACH1*	NM_004392:p.(Gly81_Ser82insAlaAla)	.	homozygous	
chr19:56088071insTCG	*ZNF787*	NM_001002836:p.(Asp366dup)	.	homozygous	
chr22:18083571T>C	*PEX26*	NM_017929:p.(Leu169Pro)	rs768604587	homozygous	25.9
chr9:66986614C>T	*SPATA31A3*	NM_001083124:p.(Arg1295Gln)	rs759025747	Heterozygous	10.16
chr9:66987043G>T	*SPATA31A3*	NM_001083124:p.(Pro1152Gln)	rs11261518	Heterozygous	11.94
chr9:66990002C>G	*SPATA31A3*	NM_001083124:p.(Ala166Pro)	rs201863232	Heterozygous	16.12

Combined Annotation Dependent Depletion (CADD), http://cadd.gs.washington.edu, accessed on 20 April 2021.

## Data Availability

The data presented in this study are openly available in ClinVar (http://www.ncbi.nlm.nih.gov/clinvar, accessed on 20 April 2021), Submission ID: SUB9433438.
